# Case report: Flail leg syndrome in familial amyotrophic lateral sclerosis with L144S *SOD1* mutation

**DOI:** 10.3389/fneur.2023.1138668

**Published:** 2023-03-22

**Authors:** Ewa Zapalska, Dominika Wrzesień, Adam Stępień

**Affiliations:** Department of Neurology, Military Institute of Medicine – National Research Institute, Warsaw, Poland

**Keywords:** flail leg syndrome, familial ALS (FALS), familial amyotrophic lateral sclerosis, SOD1 mutation, pseudopolyneuritic form, amyotrophic lateral sclerosis (ALS), L144S, phenotype of ALS/MND

## Abstract

We observed a Polish family with familial amyotrophic lateral sclerosis with heterozygous L144S *SOD1* mutation, which manifested clinically as flail leg syndrome. Flail leg syndrome is a rare phenotype of amyotrophic lateral sclerosis, with slow progression, long survival, and predominance of lower motor neuron signs at onset, as a triad of distal paresis, muscle atrophy, and hyporeflexia/areflexia, confined to the lower limbs for an extended period of time. Although familial amyotrophic lateral sclerosis is usually associated with a worse prognosis than the sporadic form of the disease, the clinical course of the disease in patients with L144S *SOD1* mutation is benign, with slow progression and long survival. This unique case report provides an in-depth clinical analysis of all of the symptomatic members of a family, who were diagnosed with amyotrophic lateral sclerosis in our clinic, including three siblings (two brothers and a deceased sister) with flail leg syndrome and their fraternal aunt, who has been previously misdiagnosed with cervical myelopathy and is living with symptoms of the disease for 15 years. Sanger sequencing of the *SOD1* gene was performed in all of the living patients, revealing an L144S (c.434T>C, p.Leu145Ser) heterozygous mutation. The aim of this case report is to increase the physician's awareness of the atypical phenotypes of amyotrophic lateral sclerosis and hopefully, to encourage further research on the factors responsible for delayed disease progression in patients with L144S *SOD1* mutation.

## 1. Introduction

Amyotrophic lateral sclerosis (ALS) is the most common motor neuron disease, which in its classic form is characterized by symptoms of simultaneous upper and lower motor neuron involvement at the onset (1). It has a mean prevalence of 5.4/100.000 in Europe (1) with flail leg syndrome (FLS, also known as pseudopolyneuritic variant) accounting for up to 5% of ALS cases (2), in which lower motor neuron signs predominate at onset, affecting the lower limbs usually as a triad of distal paresis, muscle atrophy, and hyporeflexia/areflexia (3).

Flail leg syndrome was discovered by Pierre Marie and later described by his student Patrikios in 1918 as asymmetric-onset, slowly progressing, distal lower limbs muscle weakness with the absence of lower limb deep tendon reflexes (DTRs) and presence of subtle or late upper motor neuron (UMN) signs (4). FLS is a rare phenotype of ALS, characterized by lower motor neuron (LMN) signs restricted to the lumbosacral region for an extended period of time (which varies in the literature from 12 to 24 months), with a more favorable prognosis than the classic form of ALS in the means of slower progression and longer mean survival between 75.9 and 87 months with diminished and late respiratory involvement (3). The longer the disease is confined to the lumbosacral region, the longer the survival (4).

Up to 10% of ALS cases are familial, with *SOD1* gene mutations involved in 14.8% of familial ALS cases in Europe (5). Risk factors of familial ALS include a positive family history of dementia and neurodegenerative disorders, early age of onset, predominant lower limbs symptoms, or subjective sensory signs at the onset (6, 7). Familial ALS is usually inherited in an autosomal dominant pattern, occurs on average 10 years earlier, and has a worse prognosis than sporadic ALS (8).

According to Kuzma-Kozakiewicz et al. (9), the most common *SOD1* mutation among ALS patients in Poland is K3E, followed by L144S (c.434T>C, p.Leu145Ser) which is a heterozygous, missense mutation located in exon 5 of the *SOD1* gene. The study mentioned 26 cases of L144S mutation worldwide (19 cases in Poland, 3 in Iran, 3 in Brazil, and 1 in the USA), including 10 fALS families and 3 sporadic ALS cases, out of which 66.6% of cases displayed classic ALS phenotype and in the remaining 33.3% of cases, progressive muscular atrophy was diagnosed. The clinical course of ALS in patients with L144S *SOD1* mutation is relatively benign, with slow progression and an average survival of 11 years. Initially, the symptoms are confined to the lower limbs, while bulbar symptoms appear late (9).

This case report provides a unique opportunity to analyze the clinical course of familial ALS with heterozygous L144S *SOD1* mutation in a Polish family, from the physician's point of view.

## 2. Case description

**Figure 1:**
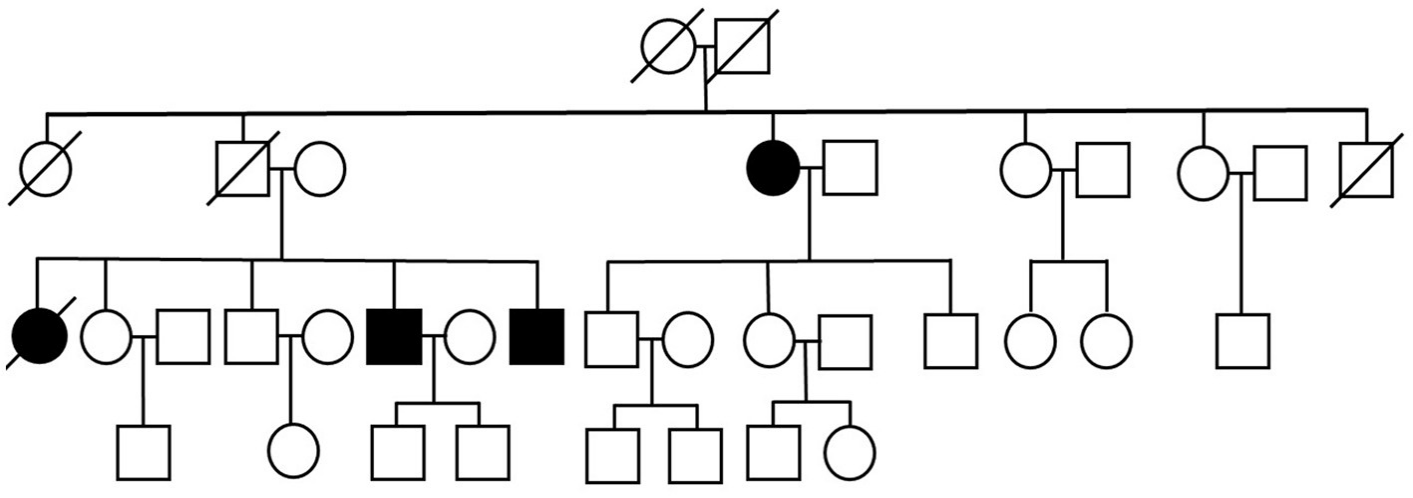
Pedigree of the studied polish family.

We present three siblings-two brothers (Case 1 and Case 3) and their deceased sister (Case 4 – retrospective analysis), with a rare phenotype of ALS (flail leg syndrome), and their fraternal aunt (Case 2), suffering from a progressive motor decline over 15 years, previously diagnosed with cervical myelopathy in another hospital and finally diagnosed with ALS in our clinic. Family pedigree has been obtained—other family members are reported to be free of any neurological and cognitive deficits (Figure 1).

Diagnostic workup of the patients included the following: laboratory blood tests, CSF analysis (including antibodies against Borrelia burgdorferi in the serum and CSF, oligoclonal bands in CSF), and MRI of the brain, cervical, and lumbosacral spine, which did not display any significant findings in all of the cases. EMG/ENG study was performed in all of the patients and significantly aided the diagnosis. All patients were diagnosed with ALS and subsequently treated with riluzole.

Sanger sequencing of the *SOD1* gene was performed in all of the living patients in 2022 (Cases 1, 2, and 3) and displayed the presence of L144S (c.434T>C, p.Leu145Ser) mutation in one allele.

The patients were not included in any other study and are presented in the order in which they were treated by the authors. The cases are summarized in Table 1.

**Table 1:** Summary of familial ALS cases described in the article.

**Case No**.	**Gender**	**Mutation/date of genetic testing**	**Age/year at symptoms' onset**	**Symptoms at onset**	**Duration of symptoms' confinement to lower limbs**	**ALS phenotype**	**Diagnostic delay**	**Time from disease onset to bulbar symptoms' onset**	**Survival**
1	M	Heterozygous L144S *SOD1* mutation (02.2022)	46 yo. (2020)	Asymmetric, distal onset lower limb paresis with predominant LMN signs and needle-like pain	2 years - ongoing	Flail leg syndrome	1,5 year	-	2 years - ongoing
2	F	Heterozygous L144S *SOD1* mutation (05.2022)	61 yo. (2007)	Asymmetric-onset, spastic paraparesis with absent patellar and Achilles DTRs	11 years	-	15 years	14 years	15 years - ongoing
3	M	Heterozygous L144S *SOD1* mutation (06.2022)	46 yo. (2017)	Asymmetric, distal onset lower limb paresis with predominant LMN signs	5 years	Flail leg syndrome	3 years	-	5 years - ongoing
4	F	N/A (deceased)	35 yo. (1998)	Distal lower limbs paresis with predominant LMN signs, needle-like thigh pain	4 years	Flail leg syndrome	4 years	5 years	12 years

### 2.1. Case 1

In February 2022, a 47-year-old man with no prior medical history was admitted to the neurological clinic due to distal lower limbs muscle weakness progressing over 2 years.

The first symptoms appeared in August 2020 in the form of a shortening of the walking distance, followed by a right foot drop in February 2021. Initially, the patient associated the symptoms with a bilateral foot injury that he suffered in 2008 as a result of a fall from a height accident.

The ENG/EMG study from October 2021 revealed features of significant axonal damage to distal segments of the tibial nerves, without signs of active denervation. After the exclusion of lumbar discopathy as a possible cause of the symptoms, the patient underwent orthopedic surgery in April 2021 (right-sided arthrodesis of the first MTP joint and talocalcaneonavicular joint)—without improvement. Since September 2021, the patient walks with a cane, suffers from trembling hands, and reports fasciculations and needle-like pain localized mostly in the lower limbs, as well as upper limbs and trunk. At the time of the patient's hospitalization, his two siblings were already diagnosed with ALS. The patient has no children.

Neurological examination on admission to the clinic revealed the following: generalized atrophy of the lower limbs muscles (more pronounced in the right calf—girth asymmetry of 3 cm) with numerous fasciculations, subtle atrophy of the left first dorsal interosseous muscle, restless tongue; asymmetric, moderate degree flaccid paraparesis (more pronounced distally, affecting the right lower limb to the greater degree than the left) with trace patellar and ankle DTRs and mildly positive Babinski sign on the left side; very brisk DTRs from the upper limbs, steppage gait.

Laboratory blood tests and CSF analysis did not reveal any significant abnormalities. MRI of the brain, cervical, and lumbosacral spine did not reveal any relevant findings. The EMG and ENG study displayed features of denervation at the C/Th and L-S spinal cord regions. Based on the clinical and electrophysiological features, probable ALS was diagnosed and riluzole was introduced. *SOD1* gene sequencing was performed, demonstrating the presence of the p.Leu145Ser variant in one allele.

### 2.2. Case 2

In May 2022, a 76-year-old bedridden woman was admitted to the neurological clinic due to progressive muscle weakness in the upper and lower limbs.

The first symptoms appeared about 15 years before as chronic back pain and walking difficulty, caused by muscle weakness affecting the left lower limb at first and then the right lower limb. The patient was walking on straight, stiff legs “as if on stilts”—any attempt to bend the knees resulted in a fall. In 2015, significant deterioration of mobility forced the patient to require the aid of a cane while walking and prompted the patient to seek inpatient neurological care (in another hospital), which resulted in a diagnosis of degenerative disc disease of the cervical and lumbar spine with cervical myelopathy and lumbar stenosis. At the time of the diagnosis, the patient claimed that her family history was positive for multiple sclerosis. In 2016, the patient underwent an L4 laminectomy, which did not result in clinical improvement. The patient did not consent to the surgical treatment of cervical myelopathy. Despite physical rehabilitation, further progression of the symptoms was observed. In addition, muscle weakness in the upper limbs is developing for approximately 3–4 years. For about a year, swallowing difficulty is occurring periodically. Currently, the patient is bedridden and uses a wheelchair with the help of others.

The patient has three children (two sons and a daughter) that are reported to be healthy. Other medical history of the patient includes: microvascular ischemic brain disease, hypertension, thyroid nodules, hypothyroidism, bilateral cataracts, a history of left tibial fracture surgery, and cholecystectomy.

Neurological examination on admission to the clinic revealed the following: bilateral atrophy of the shoulder girdle muscles, biceps and triceps brachii, lumbrical, and interossei muscles; third, fourth, and fifth fingers of both hands were fixed in a flexed position, fasciculations of the left triceps brachii muscle and hand muscles bilaterally, tongue fibrillations, minimal dysarthria, mild dysphagia, spastic paraparesis 1/5 MRC with the absence of patellar and ankle DTRs, the absence of the left plantar reflex, mildly positive Babinski sign on the right side, and paresis of the upper limbs without increased muscle tone (in the left upper limb 2/5 MRC, in the right upper limb proximally 3/5 MRC, distally 4/5 MRC) with normal DTRs.

Laboratory blood tests and CSF analysis did not reveal any significant abnormalities except for elevated TSH and ALT. MRI of the brain revealed numerous chronic microvascular changes within the pons, frontal, and parietal lobes bilaterally as well as crowding of gyri at the vertex/parafalcine region with cortico-subcortical atrophy. MRI of the cervical spine displayed multilevel degenerative disc changes with spinal and foraminal stenosis, with no evidence of cervical myelopathy. The EMG and ENG study revealed features of denervation at three spinal cord regions (C, Th, and L) and slight acute neurogenic damage at the level of the brainstem. Based on the clinical and electrophysiological features, definite ALS was diagnosed and riluzole was introduced. *SOD1* gene sequencing was performed, demonstrating the presence of the p.Leu145Ser variant in one allele.

### 2.3. Case 3

In June 2022, a 51-year-old man with ALS (diagnosed in 2020) was admitted to the neurological clinic for further diagnostic evaluation.

The first symptoms emerged in 2017 at the age of 46, when the patient started walking with a limp on the right side. The muscle weakness slowly progressed to involve the left lower limb, forcing the patient to walk with a cane from 2019 or 2020. The ENG/EMG study from March 2019 revealed features of significant axonal damage to tibial and deep fibular nerves, without traits of active denervation. From August 2019 to September 2020, the patient was hospitalized three times in the neurological clinic, which culminated in the diagnosis of ALS. Neurological examination revealed flaccid paraparesis, absence of patellar reflex on the right side, lack of ankle DTRs bilaterally, and mildly positive Babinski sign on the left side. MRI of the cervical and lumbar spine displayed degenerative disc changes with no evidence of myelopathy. Gradual progression of electrophysiological findings was observed —at first, features of denervation were present at the lumbosacral region in August 2019 and then spread to C/Th, Th, and L-S regions in September 2020. An additional EMG and ENG study from September 2021 revealed features of denervation at three spinal cord regions (C, Th, and L-S). Since January 2022, the patient walks with the support of a walking cane. The patient has two underage children.

Neurological examination on admission to the clinic revealed the following: fasciculations of deltoid muscles bilaterally, muscle atrophy of the lower limbs (most visible proximally and on the right side), minimal muscle atrophy of the right upper limb, restless tongue; asymmetric flaccid paraparesis—more pronounced in the right lower limb (paresis of hip extensors 2/5 MRC, paresis of dorsal flexors 3/5 MRC, paresis of hip flexors and plantar flexors 4/5 MRC), than in the left lower limb (4/5 MRC), lack of patellar and ankle DTRs bilaterally, lack of plantar reflex on the right side, weak plantar reflex on the left side, and discreet paresis of the right upper limb with very brisk DTRs.

Laboratory blood tests revealed mildly increased CK, decreased serum IgM levels, and impaired fasting glycemia. CSF analysis did not display any significant abnormalities except for mild elevation of IgA. MRI of the brain did not reveal any significant findings. *SOD1* gene sequencing was performed, demonstrating the presence of the p.Leu145Ser variant in one allele.

### 2.4. Case 4

The first symptoms emerged in 1998 at the age of 35, as slowly progressive distal lower limb muscle weakness with intense, needle-like pain in the thighs. The EMG study from 2001 displayed traits of axonal damage to the peripheral nerves (mainly of the lower limbs) at the level of the nerve trunks, with features of denervation and reinnervation in the muscles of the lower and upper limbs, more pronounced distally. MRI of the thoracic and lumbar spine from March 2002 did not reveal any significant findings. In July 2002, the patient was hospitalized in the neurological clinic and presented with moderate degree flaccid paraparesis and muscle atrophy (mainly distal, most pronounced in plantar flexors) with the absence of patellar and ankle DTRs and positive Babinski sign bilaterally; paresis of the fourth and fifth finger of the left hand, duck-like gait. Laboratory blood tests and CSF analysis did not reveal any significant abnormalities except for mildly elevated CK. MRI of the cervical spine was normal. The EMG and ENG study revealed features of denervation at the level of the brainstem, cervical, and lumbar spinal cord regions. Based on the clinical and electrophysiological features, probable ALS was diagnosed and riluzole was introduced.

During hospitalization in the neurological clinic in October 2003, the patient presented with tongue atrophy, mild dysarthria with discreet features of bulbar speech, flaccid quadriparesis (severe paraparesis; the weakness was more pronounced in the right limbs) with upper and lower limb muscle atrophy, trace patellar and ankle DTRs, positive Babinski sign on the left side, and mildly positive Babinski sign on the right side. The EMG and ENG study confirmed features of denervation at cervical and lumbar spinal cord regions. Last recorded neurological consultation from 2008 described flaccid quadriparesis 2/5 MRC with lower limbs areflexia. The patient died in 2011 at the age of 47 due to ALS-related respiratory failure.

## 3. Discussion

We observed that L144S (c.434T>C, p.Leu145Ser) *SOD1* heterozygous mutation in familial ALS may result in asymmetric-onset lower limb paresis (flaccid or spastic) with trace/absent lower limb DTRs, late bulbar signs, and long survival. Due to limitations of Sanger sequencing, other modifying variants within exon-distant intronic or promoter regions could not be excluded. Lower limb onset and long survival seem to be the defining features of L144S *SOD1* mutation, as depicted in Table 2, resuming the main features of all L144S *SOD1* cases described so far.

**Table 2:** Review of L144S *SOD1* cases reported in the literature.

**References**	**Population**	**Gender**	**Number of L144S *SOD1* cases/Type of mutation (if specified)**	**Age of onset**	**Site of onset**	**Clinical presentation**	**Survival (years)**
Nel et al. (10)	South African	N/A	(*n =* 1)	N/A	N/A	ALS	N/A
Gagliardi et al. (11)	Iranian		1 FALS family (*n =* 3)			ALS	
		F	Homozygous	31	Lower limb	Paresthesia of the toes, followed by painful paresis of upper and lower limbs, dysarthria, dysphagia	2
		M	Homozygous	18	Lower limb	Lower limb paresis, followed by upper limb paresis, dysphagia, respiratory failure	9
		F	Heterozygous	64	Lower limb	Asymmetric-onset, proximal paraparesis with UMN signs predominance, followed with ataxia and numbness of the lower limbs	1+
Chen et al. (12)	Chinese		1 FALS (*n =* 1), 1 SALS				
		F	Familial	50	Lower limb	LMN-dominant ALS of symmetric onset	2.6+
		F	Sporadic	48	Lower limb	Classic ALS of asymmetric onset	3.8+
Kuzma-Kozakiewicz et al. (9)	Polish	79% F, 21% M	5 FALS families (*n =* 17), 2 SALS	39.8 ± 11.0 (mean±SD) (*n =* 9)	Lower limb (*n =* 16)	50% classic ALS, 50% PMA (*n =* 8)	10.34 ± 5.77 (mean±SD) (*n =* 14)
Chadi et al. (13)	Brazilian		3 FALS families (*n =* 3)				
		F		31	Upper limb	UMN-dominant ALS	1+
		M		40	Lower limb	UMN-dominant ALS	25+
		F		22	Lower limb	UMN-dominant ALS	5+
Alavi et al. (14)	Iranian		1 FALS family (*n =* 2), 1 SALS				
		M	Heterozygous, familial	28	Lower limb	ALS	11+
		F	Heterozygous, familial	27	Lower limb	ALS	8+
		M	Heterozygous, sporadic	45	Lower limb	ALS	11+
Cudkowicz et al. (15)	US	N/A	1 FALS family (*n* = 2)	42.5 ± 10.6 (mean±SD)	N/A	ALS	12.3 ± 3.7 (mean±SD)
Sapp et al. (16)	US		1 FALS family (*n =* 2)	42.5 (*n =* 2)			
		N/A				ALS	9
		N/A				ALS	13+

Flail leg syndrome is a rare phenotype of ALS which may present with unilateral foot drop, trace deep tendon reflexes from the lower limbs, and waddling gait, which may resemble peripheral neuropathy and lead to a misdiagnosis. According to Wijesekera et al. (4) UK and Melbourne clinical studies, FLS is defined as a progressive, distal onset LMN pattern of weakness and wasting confined solely to the lower limbs for at least 12 months. Deep tendon reflexes from the lower limbs are usually diminished or absent; however, brisk lower limb DTRs or positive Babinski sign do not preclude the diagnosis of FLS, as long as hypertonia and clonus are absent (3, 4). FLS of asymmetric onset may present with brisk DTRs from unaffected limbs (8). During the disease course, the symptoms eventually spread to involve other regions and resemble classic ALS (2).

The siblings (Case 1, Case 3, and Case 4) match the criteria of FLS; however, their fraternal aunt (Case 2) with asymmetric-onset spastic paraparesis and lack of lower limb DTRs does not entirely match the aforementioned diagnostic criteria of FLS due to increased muscle tone in the lower limbs.

A longer clinical course coupled with the atypical presentation at onset may lead to a misdiagnosis, which could prolong the initiation of treatment and may lead to unnecessary interventions including surgeries and hospitalizations (8, 17). An elderly woman with a progressive disability, suffering from chronic back pain does not necessarily have to be diagnosed with cervical myelopathy or lumbar stenosis but may potentially suffer from ALS (as in Case 2), which highlights the importance of careful evaluation of patient's history, neurological examination, and electromyography.

FLS is correlated with the presence of *SOD1* mutation with an odds ratio of 3.75 (18). Therefore, in families affected by familial ALS, a detailed, at least three-generational pedigree should be obtained. Genetic testing should not be performed solely for the purpose of predicting the further clinical course of the disease, because of variable expressivity (one pathogenic variant may manifest as several different phenotypes); however, it does provide limited prognostic information. Predictive testing of minors is considered unethical. First, symptomatic individuals who are willing may undergo full gene sequencing, which is a gold standard for searching the entire coding region of the chosen gene for pathogenic variants. A positive result cannot give a definite answer to whether the individual will develop the disease due to a lack of population-based data on penetrance; however, it may be used to identify at-risk family members and to confirm the diagnosis as clinically definite laboratory-supported familial ALS in patients with clinically suspected or clinically possible ALS and in the light of emerging gene-specific therapies it gives the opportunity to participate in clinical trials (6).

Tofersen is a novel antisense oligonucleotide (ASO) drug administered intrathecally, decreasing the production of SOD1 protein in *SOD1*-ALS. Although it failed to slow down the functional decline of patients at week 28 in a phase 3 trial, it may inhibit neurodegeneration by reducing CSF neurofilament levels (19, 20).

In this case report, we have presented two generations affected by familial ALS, in which the symptoms seem to progress faster in the younger generation, possibly indicating genetic anticipation. Based on the available data from family history, it was not possible to establish the founder, who passed the mutated gene to the affected family members. In our opinion, further research studies are needed to identify the exact factors responsible for delayed ALS progression in patients with L144S *SOD1* mutation, which will certainly aid to unveil the etiology of this currently fatal disease.

## 4. Conclusion

Amyotrophic lateral sclerosis may present with varying degrees of upper and lower motor neuron involvement at the onset. Flail leg syndrome is one of the rare, atypical phenotypes of ALS with slow progression and long survival, in which lower motor neuron signs predominate for an extended period of time, affecting the lower limbs usually as distal, flaccid paresis (often of asymmetric-onset, including unilateral foot drop), muscle atrophy, and areflexia. In patients presenting lower motor neuron signs of unclear etiology, the EMG/ENG study should always be considered, which will speed up the diagnostic process and enable early treatment, including early enrollment into clinical trials. Subjective sensory symptoms such as “needle-like” pain do not preclude the diagnosis of ALS; on the contrary, they may indicate an atypical phenotype or genetic background of ALS. The role of genetic testing in ALS should be highlighted. Familial ALS with heterozygous L144S *SOD1* mutation is characterized by slow progression and extremely long survival and can manifest clinically as flail leg syndrome.

## Data availability statement

The datasets presented in this article are not readily available because of ethical and privacy restrictions. Requests to access the datasets should be directed to the corresponding author.

## Ethics statement

Ethical review and approval was not required for the study on human participants in accordance with the local legislation and institutional requirements. The patients/participants provided their written informed consent to participate in this study. Written informed consent was obtained from the individual(s) for the publication of any potentially identifiable images or data included in this article.

## Author contributions

EZ served as initiator and supervisor of the project, writer of the manuscript, responsible for the acquisition and analysis of data, treated the patients during hospitalizations, and approved the final version of the manuscript. DW was responsible for the analysis and interpretation of data, physical examination of the patients, provided editorial revisions, and approved the final version of the manuscript. AS served as substantive guidance and support, provided editorial revisions, and approved the final version of the manuscript. All authors contributed to the article and approved the submitted version.
